# *AaPKAc* Regulates Differentiation of Infection Structures Induced by Physicochemical Signals From Pear Fruit Cuticular Wax, Secondary Metabolism, and Pathogenicity of *Alternaria alternata*

**DOI:** 10.3389/fpls.2021.642601

**Published:** 2021-04-21

**Authors:** Miao Zhang, Yongcai Li, Tiaolan Wang, Yang Bi, Rong Li, Yi Huang, Renyan Mao, Qianqian Jiang, Yongxiang Liu, Dov B. Prusky

**Affiliations:** ^1^College of Food Science and Engineering, Gansu Agricultural University, Lanzhou, China; ^2^Department of Postharvest Science of Fresh Produce, Agricultural Research Organization, The Volcani Center, Bet Dagan, Israel

**Keywords:** *Alternaria alternata*, PKA protein kinase, pear fruit, wax, hydrophobicity, virulence

## Abstract

*Alternaria alternata*, the casual agent of black rot of pear fruit, can sense and respond to the physicochemical cues from the host surface and form infection structures during infection. To evaluate the role of cyclic AMP-dependent protein kinase (cAMP-PKA) signaling in surface sensing of *A. alternata*, we isolated and functionally characterized the cyclic adenosine monophosphate-dependent protein kinase A catalytic subunit gene (*AaPKAc*). Gene expression results showed that *AaPKAc* was strongly expressed during the early stages of appressorium formation on hydrophobic surfaces. Knockout mutants Δ*AaPKAc* were generated by replacing the target genes via homologous recombination events. We found that intracellular cAMP content increased but PKA content decreased in Δ*AaPKAc* mutant strain. Appressorium formation and infection hyphae were reduced in the Δ*AaPKAc* mutant strain, and the ability of the Δ*AaPKAc* mutant strain to recognize and respond to high hydrophobicity surfaces and different surface waxes was lower than in the wild type (WT) strain. In comparison with the WT strain, the appressorium formation rate of the Δ*AaPKAc* mutant strain on high hydrophobicity and fruit wax extract surface was reduced by 31.6 and 49.3% 4 h after incubation, respectively. In addition, *AaPKAc* is required for the hypha growth, biomass, pathogenicity, and toxin production of *A. alternata*. However, *AaPKAc* negatively regulated conidia formation, melanin production, and osmotic stress resistance. Collectively, *AaPKAc* is required for pre-penetration, developmental, physiological, and pathological processes in *A. alternata.*

## Introduction

*Alternaria alternata*, as a typical latent infectious fungus that can cause different diseases in more than 100 plant species (Wang et al., 2019), not only results in severe losses for the economy and the industry but also poses a potential safety hazard to animals and human beings due to the production of mycotoxins during the growth and development (Andersen et al., 2015; Estiarte et al., 2016). To date, at least seven pathotypes of *A. alternata* have been reported in several species, including tomato, strawberry, rough lemon, pear, apple, and tangerine (Thomma, 2003). Black rot of pears, caused by *A. alternata*, is the most common and severe postharvest disease (Li et al., 2007). Initially, the conidia of *A. alternata* adhere to the surface of the pear fruit, and then conidia germinate and form the appressorium at the tips of conidial germ tubes; melanin deposited in the cell wall of the appressorium is required to build up sufficient pressure to penetrate the pear surface (O’Connell et al., 2012; Fetzner et al., 2014). A wide range of exogenous physical and chemical signals such as hydrophobic surface, temperature, sugars, nitrogen sources, and fatty acids can be recognized and responded to by the fungus (Maller, 2003; Xue et al., 2008).

Plant epidermis is the first barrier to pathogen infection, and the physicochemical signals of the plant epidermis, such as hydrophobicity, waxiness, and cutinization, play an important role in affecting the infection by pathogens, especially the affinity strains (Collins et al., 2001; Gniwotta, 2005; Yoshida et al., 2015). Yoshida et al. (2015) suggested that the conidial germination and the formation of appressoria of *Blumeria graminis*, *Colletotrichum dematium*, and *Magnaporthe grisea* were significantly decreased due to reduced hydrophobicity of solid surfaces. Furthermore, in *Ustilago maydis*, appressorium formation can be efficiently induced by 12-hydroxystearic acid, 16-hydroxyhexadecanoic acid, or cutin monomers from maize leaves (Mendoza-Mendoza et al., 2009). Hexacosanal (C26-aldehyde) was found to strongly induce appressorium formation by *B. graminis* (Hansjakob et al., 2010). The C28 aldehyde was also involved in infection structure differentiation of *Puccinia graminis* (Reisige et al., 2006). These studies showed that chemical components and hydrophobicity of plant cuticular wax might activate the formation of the fungal pathogen infection structure.

In fungi, signaling pathways such as cyclic AMP-dependent protein kinase (cAMP-PKA) or mitogen-activated protein kinase (MAPK) cascades via G protein-coupled receptor recognize various exogenous signals that trigger a variety of cellular processes (Versele et al., 2001; Fredriksson et al., 2003; Liu et al., 2011). For example, Mendoza-Mendoza et al. (2009) reported that the differentiation of the infection structure in *U. maydis* induced by fatty acids or hydrophobicity requires integrity of the pheromone MAPK cascade. MAP kinase *fus* and *hog1* play positive roles in secondary metabolite synthesis of *Fusarium graminearum* and *Fusarium verticillioides* (Wang et al., 2011; Guo et al., 2016). Cyclic adenosine monophosphate (cAMP) is the key second messenger in the cAMP-PKA pathway, its concentration being regulated by both adenylate cyclase (AC) and phosphodiesterase (Karin et al., 2013). Activation of AC leads to the formation of the second messenger cAMP, which is subsequently bound by the regulatory subunits of the protein kinase A (Zhu et al., 2017). The 3′,5′-cyclic adenosine monophosphate-dependent protein kinase A (PKA), consisting of two regulatory subunits and two catalytic subunits, is a serine/threonine protein kinase. In general, binding of cAMP to the regulatory subunits results in the detachment and activation of the catalytic subunits (Kim et al., 2005).

The cAMP-PKA signaling pathway is involved in a range of physiological processes in fungi, including growth, infection structure differentiation, cell wall integrity, stress responses, virulence, and secondary metabolism (Lengeler et al., 2000; Nadal et al., 2010; Fuller and Rhodes, 2012). In *U. maydis*, only *Adr1* kinase activity is involved in the dimorphic transition and pathogenesis (Durrenberger et al., 1998). In *Verticillium dahlia*, PKA actively regulates ethylene biosynthesis (Tzima et al., 2010). In *Cryptococcus neoformans*, deletion of adenylyl cyclase *cac1* caused production of defective mating hyphae and failure in melanin production (Alspaugh et al., 2002). The cAMP-PKA signal pathway has also been found to positively regulate the growth, spore germination, and pathogenicity of some fungi such as *Colletotrichum lagenarium*, *Colletotrichum trifolii*, *Colletotrichum gloeosporioides*, *Magnaporthe oryzae*, and *Candida albicans* (Cloutier et al., 2003; Yamauchi et al., 2004; Priyatno et al., 2012), which indicates that the conservative cAMP-PKA signal pathway is maintained in the regulation of biological processes. However, several studies also reported that the function of the cAMP-PKA pathway in regulating secondary metabolism occurred in a species-specific and metabolite-specific dependent manner (Yu and Keller, 2005; Choi and Xu, 2010). In the adenylate cyclase *Fac1* deletion mutant of *F. verticillioides*, the synthesis of fumonisin is normal, but the content of bikaverin increases (Choi and Xu, 2010). In *Aspergillus nidulans*, PKA negatively regulates the biosynthesis of the secondary metabolite sterigmatocystin (Yu and Keller, 2005). In addition, it has been reported that PKA plays an important role in the regulation of light responses in *Trichoderma atroviride* and *A. nidulans* (Shimizu and Keller, 2001; Casas-Flores et al., 2006), and phosphorylation of PKA could activate transcription factors necessary for light-responsive genes. In *Saccharomyces cerevisiae*, PKA is activated in a favorable external nutritional environment (glucose/nitrogen source) and causes yeast cells to show increased sensitivity to various stresses (Hasan et al., 2002).

Although many studies have been done to elucidate PKA catalytic subunits in other fungi, the specific roles of PKA catalytic subunits on the infection structure and pathogenicity remain largely unknown in the process of recognition by *A. alternata* and its response to the physicochemical signals of pear fruit cuticular wax. In this study, we generated and characterized the deletion of PKA catalytic subunits (*AaPKAc*) in *A. alternata*, and *AaPKAc* genetic complementation strain (*AaPKAc-c*) was constructed. Overall, our results demonstrate that the deletion of *AaPKAc* mutant increased intracellular cAMP content but decreased PKA content. The formation of appressoria and infection hyphae was reduced in the Δ*AaPKAc* mutant strain. Although the Δ*AaPKAc* mutant still recognized and responded to high surface hydrophobicity and different wax surface, its recognition ability was significantly lower than that of the WT strain. In addition, *AaPKAc* is required for the hyphal growth, biomass, pathogenicity, and toxin production. However, *AaPKAc* negatively regulates conidia formation, melanin production, and osmotic stress resistance. These findings provide the basis for the further understanding of the importance of *AaPKAc* in the pathogenic differentiation of *A. alternata*.

## Materials and Methods

### Fruit and Chemical Sources

Fruits of “Zaosu” pear (*Pyrus bretschneideri* Rehder) were picked from the Tiaoshan Farm in Jingtai County, Gansu Province, China. Healthy pears of uniform size, with no mechanical damage, were selected, and the fruits were individually packaged in a plastic mesh bag, transported the laboratory, and stored at 4°C.

### Pathogens

The wild-type strain (WT) was originally isolated from naturally decayed Zaosu pear fruit and characterized as *Alternaria alternata* (KY397985.1), incubated on potato dextrose agar medium (PDA) at 28°C and used as the recipient strain for genetic modifications. The method described by Tang et al. (2017) was applied to a spore suspension of *A. alternata* with a final concentration of 1 × 10^5^ spores mL^–1^.

### Vector Construction and Transformation

The genomic DNA of *A. alternata* WT strain was extracted using genomic DNA purification kit (Axygen, United States) according to the manufacturer’s protocols. The pCAMBIA1300-HPH (*pCHPH*) vector was donated by the Chinese Academy of Sciences. To characterize *AaPKAc* (XP 018391379.1) genes, the gene replacement vectors *AaPKAc-up-pCHPH-AaPKAc-down* were generated as described by Ma et al. (2017). The 5′ (about 1,000 bp) and 3′ (about 1,000 bp) flanks of the ORF of each gene were amplified from genomic DNA of the WT strain by PCR with primer pairs (Supplementary Table 1). The 5′ and 3′ fragments of each gene were then cloned into the upstream and downstream of *pCHPH* vector respectively by the homologous recombination method (Supplementary Figure 1), using Gibson Assembly Master Mix kit (New England Biolabs, MA, United States) according to the manufacturer’s instructions. This procedure resulted in gene replacement vectors that had the selective marker hph gene flanked by the ORF flanking sequences from each of the genes. *Agrobacterium*-mediated transformation was performed as previously described (Wei et al., 2016). Transformants were transferred to PDA plates containing 80 μg mL^–1^ of hygromycin B (Roche, Mannheim, Germany) for a second round of selection. PCR assays with the specific primer pairs were performed with the genomic DNA, and the correct transformants were selected for further quantitative RT-PCR (qRT-PCR) analysis (Supplementary Figure 2).

In genetic complementation according to a previously described method (Tsai et al., 2013), the *AaPKAc* ORF was amplified using a high-fidelity DNA polymerase and cloned into the expression vector pBARGPE1 (Miaolin Biotechnology Co., Ltd., Wuhan, China) under the control of the gene promoter gpdA and gene terminator trpC. Then, the plasmid was transformed into Δ*AaPKAc* using the PEG-mediated transformation method. Transformants were transferred to PDA plates containing 50 μg mL^–1^ of bialaphos (Shanghai Yuanye Biotechnology Co., Ltd.) for a second round of selection and further confirmation by PCR and qRT-PCR analysis.

### Quantitative RT-PCR Analysis of Gene Expression

Conidia suspensions (5 × 10^5^ spores mL^–1^) were placed onto a hydrophobic film for different time periods. Total RNA was extracted from 5 × 10^5^ conidia using TRIzol reagent (QIAGEN, Shanghai, China) according to the manufacturers’ protocol. Reverse transcription was performed using 2 μg of RNA. GAPDH was used as an internal control. For qRT-PCR analysis, amplifications were performed using a Bio-Rad CFX96 real-time thermal cycler and QIAGEN QuantiNova^TM^ SYBR^®^ Green PCR Kit. Three replicates were performed for each sample, and the relative gene expression levels were calculated using the 2^–△△*ct*^ method as described by Livak and Schmittgen (2001). The primers shown in Supplementary Table 1 were used for PCR reactions.

### Extraction of Fruit Wax

Fruit wax was extracted using the method of Tang et al. (2017). “Pingguo” pear fruits were washed with tap water and air dried, immersed in a 1000-mL beaker containing 600 mL of chloroform and agitated twice at room temperature for 30 s. The extract was filtered through eight layers of gauze and transferred to a distillation flask, and the solvent was removed by vacuum distillation (water temperature 40°C). The obtained pear fruit cuticular wax extract was refrigerated at 4°C for subsequent experiments.

### Determination of cAMP and PKA Contents

The mycelia of the WT strain, Δ*AaPKAc* mutant strain, and AaPKAc-c strain were quickly frozen and ground with liquid nitrogen, and 1 mL PBS buffer (pH 7.4) was added. After centrifugation at 3,000 rpm for 20 min at 4°C, the supernatant was collected. The contents of cAMP and PKA were measured by cAMP Complete ELISA Kit and PKA Complete ELISA Kit (Meilian Biotechnology Co., Ltd., Shanghai, China); three replicates were conducted for each strain.

### Spore Germination and Appressorium Formation Assays

#### *In vitro* Assays

A GelBond hydrophobic film (Youningwei Biotechnology Co., Ltd., Shanghai, China) (contact angle 74.63°) was cut into 5 cm × 2 cm rectangles. In the first treatment group, rectangles were coated with 20 μL (contact angle 100°) or 100 μL (contact angle 108°) pear fruit cuticular wax, then placed on a clean glass slide. In the second treatment group, rectangles were coated with either 20 μL paraffin wax, 40 μL fruit wax, or 60 μL beeswax; samples were placed onto clean slides to ensure hydrophobicity (contact angle of 101°). A 20-μL spore suspension (1 × 10^5^ spores mL^–1^) of WT strain, Δ*AaPKAc* mutant strain, or AaPKAc-c strain was pipetted onto the treated hydrophobic film rectangles, which were then incubated at 28°C and 95% relative humidity. Each treatment comprised three replicates. The spore germination rate and appressorium formation rate were calculated after 2, 4, 6, and 8 h of incubation (Belding et al., 2000).

#### *In vivo* Assays

Peel segments (3 cm × 3 cm × 1 cm) of intact and dewaxed “Zaosu” pear fruit were inoculated with 1 × 10^5^ spores mL^–1^ suspension of the WT strain, Δ*AaPKAc* mutant strain, or AaPKAc-c strain. Following the method of Hansjakob et al. (2010), all pear peel segments were placed in petri dishes with 3MM filter paper moistened with sterile water and incubated at 28°C in the dark. After 0, 6, 9, and 12 h of incubation, peel segments were placed onto 3MM filter paper moistened with a 3:1 ethanol: acetic acid (v/v) solution for 7 h with inoculated surfaces up. Samples were then transferred to filter paper moistened with lactoglycerol (1: 1: 1, lactic acid: glycerol: water, v/v/v) for 3 h, and then fungal structures were stained with lactophenol cotton blue for 5 min. The experiment was repeated three times. Pre-penetration processes were evaluated as *in vitro* assays.

### Growth and Development Phenotype Analysis

A 2-μL spore suspension of WT strain, Δ*AaPKAc* mutant strain, and AaPKAc-c strain was inoculated in the center of PDA covered with sterile cellophane film under sterile conditions, cultured at a constant temperature of 28°C for 4 days, and removed and the cellophane film weighed.

Mycelial growth and spore production were detected following previously described methods (Xu et al., 2018). A 2-μL spore suspension of WT strain, Δ*AaPKAc* mutant strain, or AaPKAc-c strain was inoculated in the center of the PDA medium and cultured at a constant temperature of 28°C and photographed and the diameter of the colony measured every day. Samples from each medium were collected with 10 mL of sterile water after 7 days of incubation, and the spore concentration counted using a hemacytometer, with three replicates per treatment.

### Evaluation of Stress Response

The tolerance of WT strain, Δ*AaPKAc* mutant strain, and AaPKAc-c strain to multiple stresses was performed. The spore suspension was inoculated on PDA containing either 1 mol L^–1^ NaCl, 1 mol L^–1^ sorbitol for osmotic stress, or 3 mmol L^–1^ H_2_O_2_ for oxidative stress, and PDA was used as control. The experiment was repeated three times. The growth inhibition rate (%) of stress to the mutant and WT strains was analyzed by measuring the colony diameters as previously described (Wei et al., 2016). Inhibition rate = (P - T)/P × 100%. Among them, P represents the colony diameter of PDA medium, and T represents the colony diameter in response to different stresses.

### Determination of Virulence

The *in vivo* assay was carried out according to a previously described method with minor modifications (Li et al., 2016). “Zaosu” pears were dipped into 2% sodium hypochlorite for 30 s, washed with distilled water and air-dried at room temperature, and wounded on the epidermis in the equatorial region with a sterile punch (2 mm deep, 5 mm wide), and each wound site was inoculated with 20 μL of spore suspension (1 × 10^6^ spores mL^–1^) of the WT strain, Δ*AaPKAc* mutant strain, or AaPKAc-c strain; each strain required nine pear fruits. Fruits were incubated in plastic boxes with 55% relative humidity at room temperature (23 ± 1°C). Disease severity was determined 1, 3, 5, and 7 days after the treatment by measuring the lesion diameter.

### Determination of Melanin Content

The WT strain, Δ*AaPKAc* mutant strain, and AaPKAc-c strain were cultured in PDA at 28°C for 4 days, followed by grinding in liquid nitrogen of accurately weighed 0.5 g of fungal mycelium. The ground material was placed in a conical flask containing 30 mL of 1 mol L^–1^ NaOH and extracted in a boiling water bath for 5 h, shaking once every 30 min, paying attention to supplementing NaOH, cooled at 20°C for 20 min, and filtered with double filter paper. The filtrate was adjusted to pH 2.0 with 7 mol L^–1^ HCl and centrifuged at 10,000 × *g* for 15 min to obtain a crude extract of melanin.

The crude extract of melanin was added with 5 mL 7 mol L^–1^ HCl, mixed well, boiled in a water bath for 2 h, and centrifuged at 10,000 × *g* for 15 min. The precipitate dissolved with 1 mol L^–1^ NaOH, and the resulting solution adjusted to pH 2.0 with 7 mol L^–1^ HCl and centrifuged at 10,000 × *g* for 15 min; this step was repeated three times. The precipitate was made up to volume 30 mL with 1 mol L^–1^ NaOH solution. As blank, 1 mol L^–1^ NaOH was used. The calibration curve was done with melanin (Sigma-Aldrich Co., Ltd., Shanghai, China) standard solutions with concentrations of 0, 10, 20, 30, 40, 50, and 60 mg L^–1^. Absorbance of the solution was measured at 400 nm with an ultraviolet spectrophotometer (UV-2450, Daojing, Japan). The experiment was repeated three times.

### Mycotoxin Extraction and HPLC-MS Analysis

The mycotoxin extraction followed Wang et al. (2016) with some modifications. Alternariol (AOH), alternariol monomethyl ether (AME), altenuene (ALT), and tentoxin (TEN) were purchased from Pribolab (Pte. Ltd., Singapore) and stored at −20°C for HPLC analysis. The WT strain, Δ*AaPKAc* mutant strain, and AaPKAc-c strain were cultured in PDA at 28°C for 4 days, followed by grinding in liquid nitrogen of accurately weighed 0.5 g of fungi mycelium and transferring the hyphae to 10 mL sterile centrifuge tubes, Subsequently, 2.5 mL of acetonitrile and water extract containing 0.3% formic acid (4:1 v/v) was added. The solution was vortexed and was extracted at 150 rpm at room temperature for 30 min. Then, 0.25 g anhydrous MgSO_4_ and 0.04 g NaCl were added, and the mixture was shaken vigorously for 1 min, followed by centrifugation at 10,000 rpm for 10 min at 4°C. Finally, the supernatant was taken through a 0.22-micron organic filter, quasi-determined to 1.2 mL, and the sample was prepared for HPLC analysis. Separation and qualitative analysis of TEN, AOH, AME, and ALT were performed using a mass spectrometer (Agilent 1290, Anjielun, Shenzhen, China) equipped with the electrospray ionization (ESI) source.

HPLC conditions were as follows: column, C18 (250 mm × 4.6 mm, 5 μm); column temperature, 35°C; injection volume, 5 μL; mobile phase A, deionized water; mobile phase B, methanol. Gradient elution conditions were after 70% retention for 1 min; gradient elution conditions are shown in Supplementary Table 2. The mass spectrometric parameters such as monitoring ion, cone voltage, and collision voltage of four *Alternaria* species are shown in Supplementary Table 3 (Zhang et al., 2020).

### Statistical Analysis

Determinations were replicated at least three times. Mean and standard error (±SE) of all data were calculated by Microsoft Office 2010, and SPSS 19.0 (SPSS, Inc., Chicago, IL, United States) was used to perform ANOVAs and Duncan’s multiple-range tests at a 5% level.

## Results

### Characterization of the cAMP-Dependent Protein Catalytic Subunits of Gene *AaPKAc*

*AaPKAc* was found to contain a 1571-bp open reading frame interrupted by two small introns of 59 and 49 bp, which encodes a polypeptide of 488 amino acids. Sequence alignment and phylogenetic analysis revealed that *AaPKAc* is highly conserved among fungi. The *AaPKAc* gene belongs to the same evolutionary branch as the fungi, such as *Setosphaeria turcica* and *Bipolaris oryzae*, with approximately 88% homology with the catalytic subunits of PKA in *S. turcica* (KC521478.1) (Supplementary Figure 3A). The deduced amino acid sequences of *AaPKAc* indicated that *AaPKAc* possesses conserved recognition sequence of catalytic subunits Arg (R)-X-Ser/Thr (S/T)-Y, among which Arg plays an important role in the substrate recognition of kinases (Supplementary Figure 3B).

**FIGURE 1:**
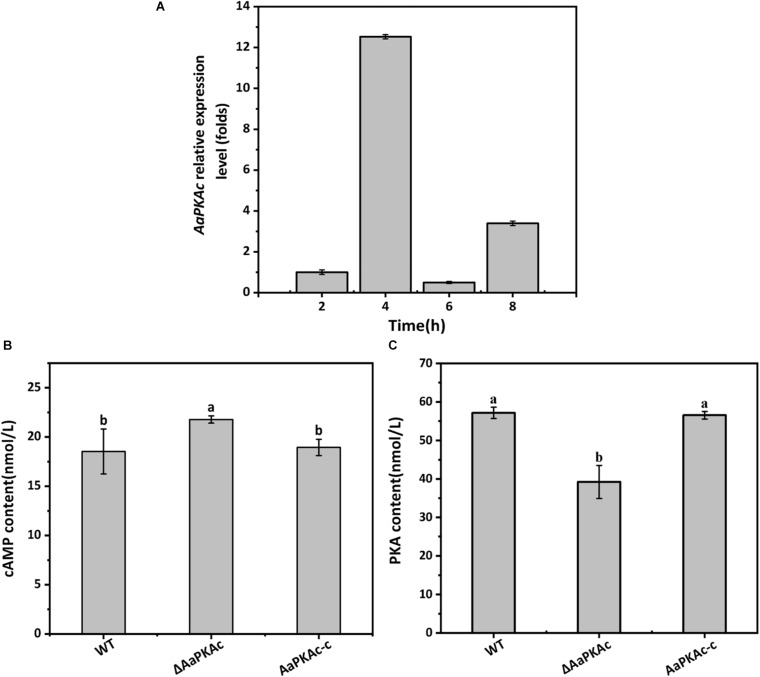
The gene expression of *AaPKAc* on the hydrophobic surface at the spore germination (2 h), appressorium formation (4 h), bud tube elongation (6 h), and infection hyphae formation (8 h) stage **(A)**. GAPDH was used as an internal control; three replicates were performed for each sample. Intracellular cAMP **(B)** and PKA **(C)** contents of the wild-type strain (WT), the Δ*AaPKAc* mutant, and *AaPKAc* genetic complementation strain (AaPKAc-c) were analyzed. Mycelia were harvested after 7 days of incubation on PDA. Vertical lines indicate standard error (±SE); different letters indicate significant differences among treatments (*P* < 0.05).

Gene expression analysis showed that *AaPKAc* was strongly expressed during the early stage of appressorium formation on the hydrophobic surface (4 h after incubation), which was 12-fold higher than at the spore germination stage (2 h after incubation) (Figure 1A).

### Deletion of AaPKAc Affects cAMP and PKA Contents of *A. alternata*

Strain Δ*AaPKAc* had 18% higher intracellular cAMP levels than the WT strain (Figure 1B). Intracellular PKA content of Δ*AaPKAc* was significantly decreased by 31.4% relative to the WT strain (Figure 1C). The AaPKAc-c strain showed the same cAMP and PKA production as the WT strain.

### AaPKAc Is Involved in Infection Structure Differentiation Induced by Physicochemical Cues From the Pear Fruit Surface

#### *In vitro* Test

**FIGURE 2:**
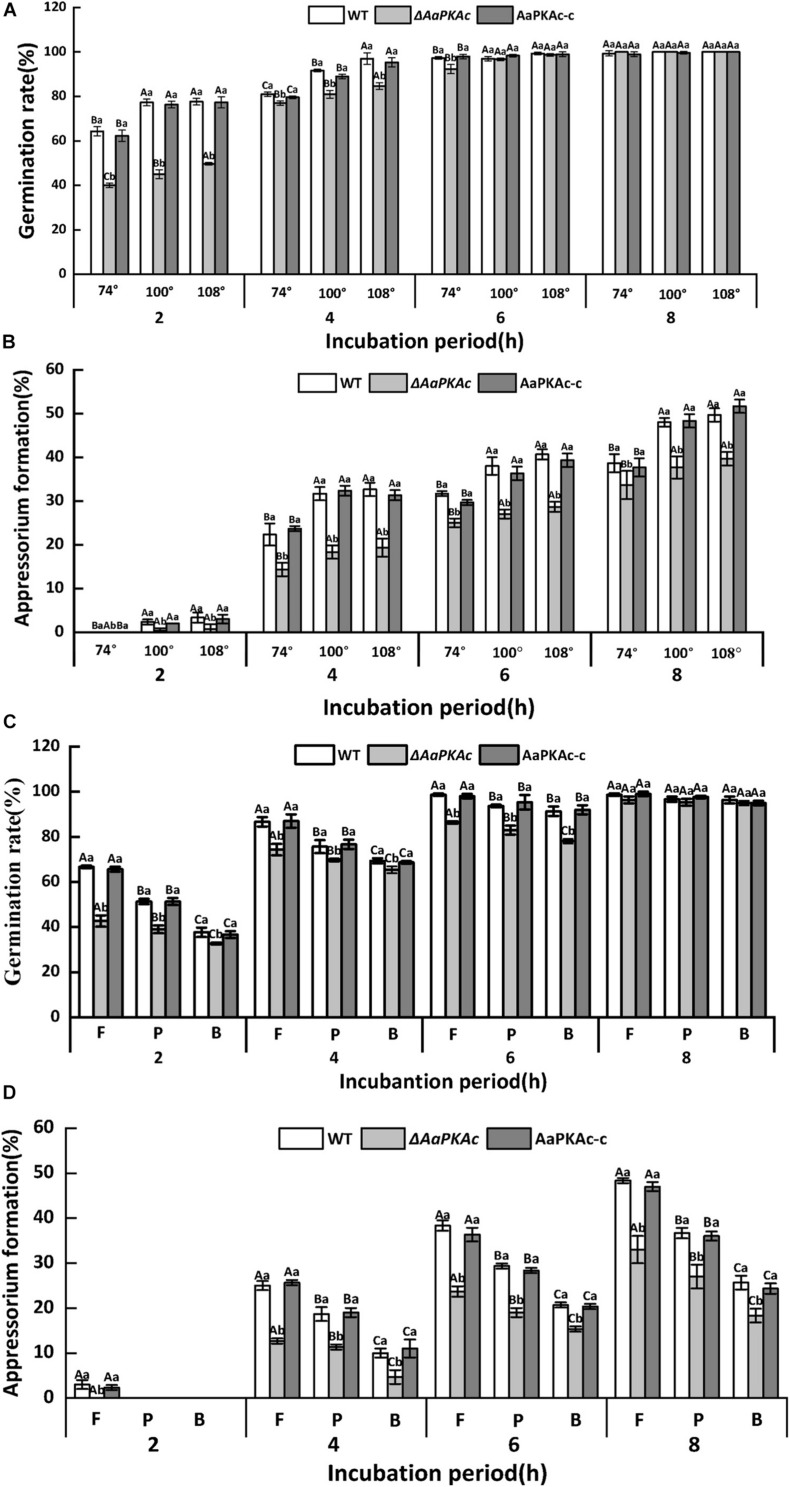
Effect of *AaPKAc* on spore germination rate **(A,C)** and appressorium formation rate **(B,D)** of the wild-type strain (WT), the Δ*AaPKAc* mutant, and *AaPKAc* genetic complementation strain (AaPKAc-c) induced by different hydrophobic surfaces (74°, 100°, 108°) and fruit wax (F), paraffin (P), and beeswax (B) coating surface. The vertical line indicates a standard error (±SE). Uppercase letters indicate differences among different contact angles; lowercase letters indicate the same difference in contact angle among treatments (*P* < 0.05).

The hydrophobicity of the surface of the GelBond film significantly increased with increasing amounts of fruit wax (Table 1). As shown in Figure 2, coating the GelBond film with fruit wax extract significantly promoted the spore germination rate and appressorium formation rate of *A. alternata*. The *AaPKAc* deletion mutant Δ*AaPKAc* significantly reduced the germination rate and appressorium formation rate (*P* < 0.05). The germination rate and appressorium formation rate of the Δ*AaPKAc* mutant strain on highly hydrophobic surfaces (108°) were decreased by 5 and 31.6% compared with the WT strain after 4 h of cultivation, respectively. The deletion of *AaPKAc* has a more significant effect on the reduction of the appressorium formation rate. The germination rate and appressorium formation rate of the AaPKAc-c strain were comparable to the WT strain (Figures 2A,B). In addition, although the Δ*AaPKAc* mutant still recognized and responded to a high hydrophobicity surface, its recognition ability was significantly lower than that of the WT strain.

**TABLE 1:** Hydrophobicity of the gelbond hydrophobic membrane coated with different contents of pingguo pear wax extract.

**Treatment**	**Gelbond hydrophobic film**	**20 μL fruit wax-coated Gelbond hydrophobic film**	**100 μL fruit wax-coated Gelbond hydrophobic film**
Contact angle (°)	74.63 ± 1.24	100.23 ± 0.11	108.35 ± 0.47

The response of *A. alternata* to the coating surface of fruit wax, paraffin, and beeswax with the same hydrophobicity (contact angle of 101°) was studied. In comparison with the paraffin (P) and beeswax (B), fruit wax (F) significantly induced the spore germination and appressorium formation rates of *A. alternata* (Figure 2). The *AaPKAc* deletion mutant Δ*AaPKAc* showed a significantly reduced germination rate and appressorium formation rate (*P* < 0.05). In the strains carrying a defective *AaPKAc* locus, the germination rate decreased on the fruit wax surface as much as 36.4% compared to the WT strain after 2 h incubation (Figure 2C). The Δ*AaPKAc* mutant strain has a more significant effect on the reduction of appressorium formation rate compared to spore germination rate: the appressorium formation rate of Δ*AaPKAc* on fruit wax surface was decreased by 49.3% compared to WT strain after 4 h incubation (Figure 2D). The AaPKAc-c strain displayed the spore germination and appressorium formation rate of the WT strain. Similarly, although the Δ*AaPKAc* mutant still recognized and responded to different surface waxes, its recognition ability was significantly lower than that of the WT strain.

#### *In vivo* Test

**FIGURE 3:**
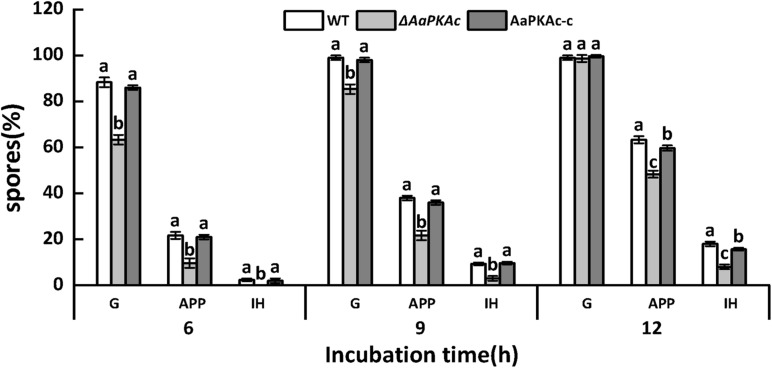
Effect of *AaPKAc* on spore germination rate (G), appressorium formation rate (APP), and infection hyphae (IH) of the wild-type strain (WT), Δ*AaPKAc* mutant, and *AaPKAc* genetic complementation strain (AaPKAc-c) on the pear epidermis. Vertical lines indicate standard error (±SE); different letters indicate significant differences among treatments (*P* < 0.05).

In order to further evaluate the effect of *AaPKAc* on infection hyphae of *A. alternata*, the infection structure of the WT strain, Δ*AaPKAc* mutant, and AaPKAc-c strain on the pear epidermis was observed. As shown in Figure 3, 6 h after incubation the spore germination, the rate of the Δ*AaPKAc* mutant strain was reduced 28.4% compared with the WT strain. However, 12 h after incubation there were no significant differences in spore germination rates between WT strain, Δ*AaPKAc* mutant, and AaPKAc-c strain which were almost up to 100%. In comparison with the WT strain, the appressorium formation and infection hyphae of Δ*AaPKAc* were decreased by 23.8 and 55.6%, respectively. The defects in spore germination, appressorium formation, and infection hyphae were rescued in AaPKAc-c strain.

### *AaPKAc* Is Involved in the Development and Stress Tolerance of *A. alternata*

**FIGURE 4:**
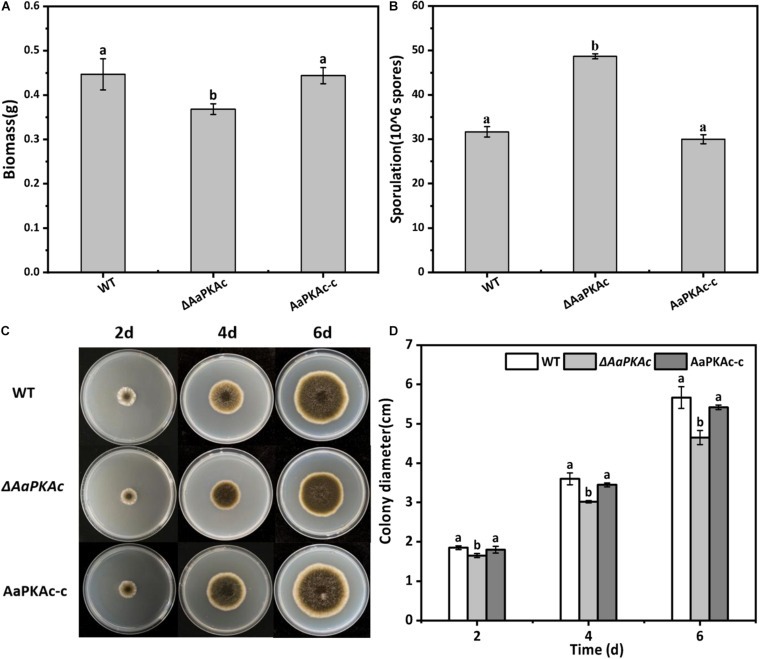
Effect of *AaPKAc* on biomass **(A)**, spore production **(B)**, mycelial growth **(C)**, and colony diameter **(D)** of *A. alternata.* A 2-μL spore suspension of the wild-type strain (WT), the Δ*AaPKAc* mutant strain, or *AaPKAc* genetic complementation strain (AaPKAc-c) was inoculated in the center of the PDA medium and cultured at a constant temperature of 28°C and photographed and the diameter of the colony measured every day. Treatments followed by different letters are significantly different according to Duncan’s multiple-range test (*P* < 0.05).

**FIGURE 5:**
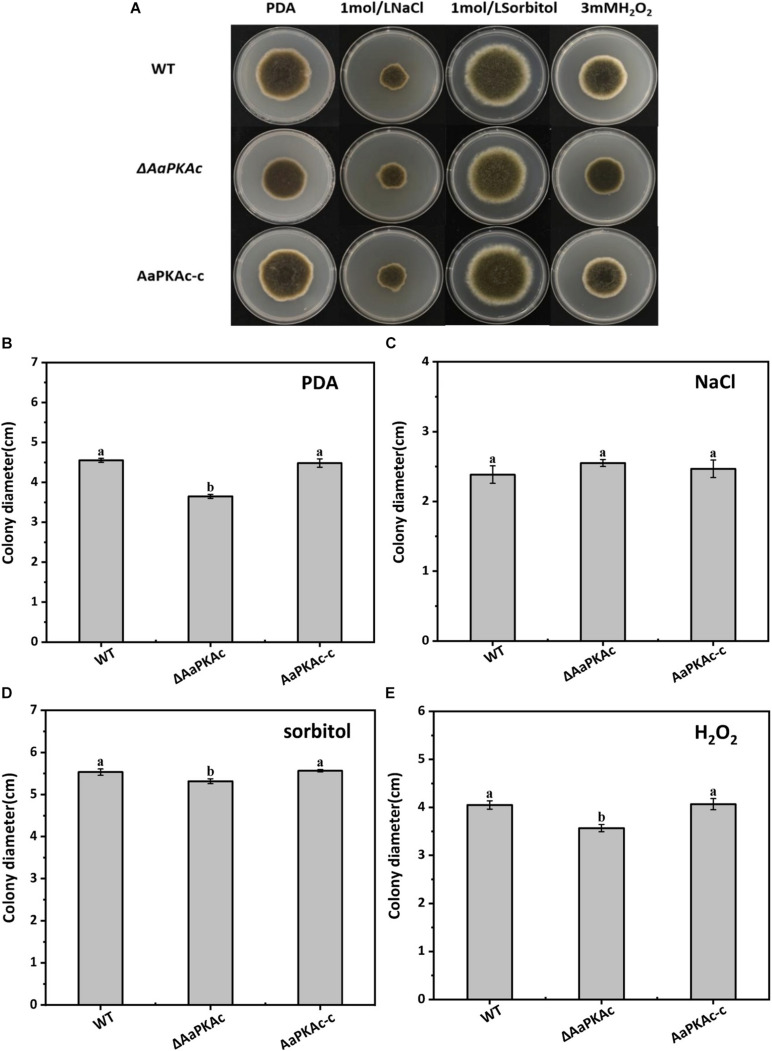
Effect of *AaPKAc* on osmotic stress and oxidative stress tolerance. Images of the wild-type strain (WT), Δ*AaPKAc* mutant, and *AaPKAc* genetic complementation strain (AaPKAc-c) were taken after 5 days of incubation on PDA supplemented with 1 mol L^–1^ NaCl, 1 mol^–1^ sorbitol, or 3 mM H_2_O_2_ **(A)**. Colony diameters of *A. alternata* were determined after 5 d of incubation on PDA **(B)** supplemented with 1 mol L^–1^ NaCl **(C)**, 1 mol^–1^ sorbitol **(D)**, or 3 mM H_2_O_2_ **(E)**.

Deletion of *AaPKAc* reduced biomass by 18% compared with the WT strain (Figure 4A). However, more conidia were observed in Δ*AaPKAc* mutants 7 days after incubation, which significantly increased by 55 and 62%, respectively, compared with the WT strain and the complementation strain (Figure 4B). After testing the growth rate of mycelia from each strain, the Δ*AaPKAc* mutant grew slower than the WT or complemented strains cultured on the PDA medium (Figures 4C,D). The growth of Δ*AaPKAc* mutants on the PDA medium with compounds known to cause different stresses was determined. Under the treatment of 1 mol L^–1^ NaCl, the colony diameter inhibition rate of the WT strain and AaPKAc-c strain was 43.63 and 45.01%, respectively, while only 35.44% inhibition rate took place in the Δ*AaPKAc* mutant strain (Figures 5B,C). Similarly, the inhibition rate of the Δ*AaPKAc* mutant strain was reduced compared with the WT strain and AaPKAc-c strain under the treatment with 1 mol L^–1^ sorbitol, indicating that deletion of *AaPKAc* increased tolerance to hyperosmotic stress compared with the WT strain (Figures 5A,D). No obvious change in H_2_O_2_ stress tolerance was detected between the Δ*AaPKAc* strain and WT strain (Figures 5A,E), indicating that Δ*AaPKAc* is not involved in the response of *A. alternata* to oxidative stress.

### *AaPKAc* Is Required for Full Virulence of *A. alternata*

**FIGURE 6:**
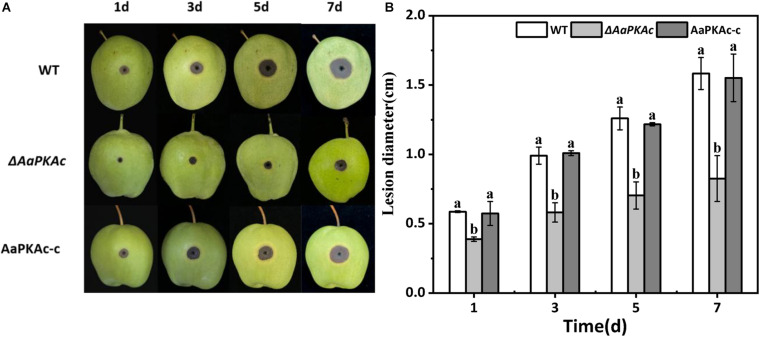
Effect of *AaPKAc* on the pathogenicity of *A. alternata.* Fungal pathogenicity was assayed on pear fruits; each “Zaosu” pear fruit was wounded on the epidermis in the equatorial region with a sterile punch (2 mm deep, 5 mm wide); each wound site was inoculated with 20 μL of conidial suspensions (10^6^ spores mL^–1^) prepared from the wild-type (WT), Δ*AaPKAc* mutant, or *AaPKAc* genetic complementation strain (AaPKAc-c) **(A)**. Each strain required nine pear fruits; a total of 27 pears were counted. Statistical analysis of lesion diameter shown as histograms **(B)**. The vertical line indicates standard error (±SE). Different letters denote significant differences (*P* < 0.05).

Fungal pathogenicity was assessed by inoculating conidial suspensions on “Zaosu” pear fruit. As shown in Figure 6, compared with pear fruits inoculated with the WT strain, the lesion diameter of the pear fruit inoculated with the Δ*AaPKAc* mutant strain was obviously reduced. The lesion diameter of the pear fruit inoculated with the Δ*AaPKAc* mutant strain was only 37% in the pear fruits inoculated with the WT strain at 5 days after inoculation. The AaPKAc-c strain induced lesion formation at rates comparable to those caused by the WT strain.

### *AaPKAc* Plays Both Positive and Negative Roles on the Secondary Metabolites

**FIGURE 7:**
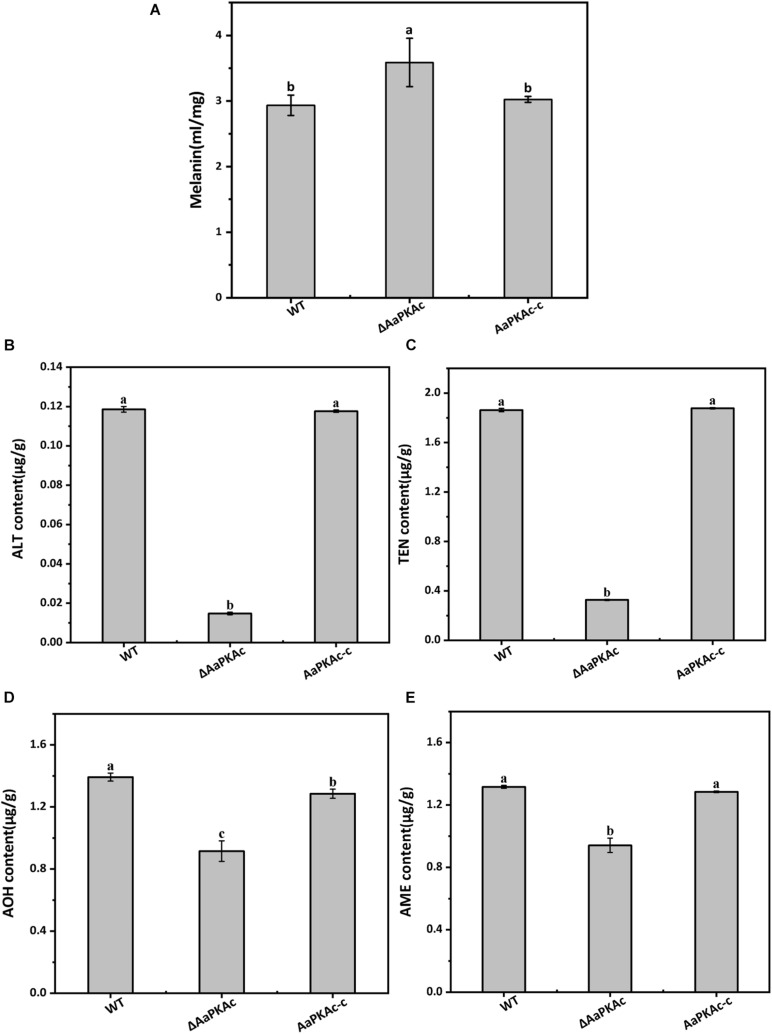
Effect of *AaPKAc* on melanin and mycotoxin production. The wild-type strain (WT), Δ*AaPKAc* mutant strain, and *AaPKAc* genetic complementation strain (AaPKAc-c) were cultured in PDA at 28°C for 4 days. The melanin content of the WT, Δ*AaPKAc* mutant, or AaPKAc-c strain was detected **(A)**. Contents of alternariol (AOH) **(B)**, alternariol monomethyl ether (AME) **(C)**, altenuene (ALT) **(D)**, and tentoxin (TEN) **(E)** in the WT, Δ*AaPKAc* mutant, or AaPKAc-c strain. Bars indicate the standard errors (±SE). Different letters denote significant differences (*P* < 0.05).

To further confirm the possible mechanism of *AaPKAc* deletion on the pathogenicity of *A. alternata*, the content of melanin was extracted and detected. The result showed that the melanin content of Δ*AaPKAc* mutant strains increased by 22% compared with the WT strain (Figure 7A). Furthermore, four mycotoxins including TEN, AME, AOH, and ALT were extracted and detected by HPLC-TOF-ESI-MS in the mycelium of the WT strain, Δ*AaPKAc* mutant, and AaPKAc-c strains. In comparison with the WT strain, Δ*AaPKAc* significantly reduced mycotoxin production; the TEN, AME, AOH, and ALT contents of the Δ*AaPKAc* mutant strain were dramatically decreased by 11, 29, 34, and 88%, respectively, compared with the WT strain. Melanin and toxin production of AaPKAc-c was comparable to those of the WT strain (Figures 7B–E).

## Discussion

The cAMP-dependent protein kinase PKA is a serine/threonine kinase, which catalyzes the transfer of γ-phosphoryl from ATP to serine/threonine residues of protein substrates. The inactive PKA holoenzyme is a heterotetramer composed of two catalytic subunits and regulatory subunits (Tsai et al., 2013; Selvaraj et al., 2017). The catalytic subunit of PKA is well conserved in structure and function. The results presented in this study showed that the functional domains of *AaPKAc* have high homology with PKA catalytic subunits of *S. turcica* (Supplementary Figure 3A) and contain the ATP-binding site, polypeptide substrate site, and contact site of the regulatory subunit.

In response to extracellular stimuli, the fungus produces multiple point sources of cAMP throughout the cell, resulting in the gradual accumulation and increase of the basal or steady-state level of cAMP. On attaining a critical threshold concentration, cAMP can further activate several important effectors, the most important being PKA (Ramanujam and Naqvi, 2010). In *M. oryzae*, the reduced or lack of PKA activities results in an increase in intracellular cAMP (Li et al., 2017). The data presented in this study also showed that the deletion of the *AaPKAc* gene increased the intracellular cAMP level; this may be due to the decreases of *AaPKAc* binding. A range of environmental cues and cellular signals that modulate the infectious morphogenesis of plant-pathogenic fungi such as hardness, hydrophobicity, host surface chemicals, waxes, and ethylene have been identified, which were important determinants of the formation of infection structures (Ahn and Suh, 2007). The results of the present study showed that the spore germination and appressorium formation of *A. alternata* were significantly induced as the surface hydrophobicity increased (Figures 2A,B). Similar results were also found when the hydrophobicity of wax on barley leaves induced the mycelial expansion and appressorium formation in *B. graminis* (Zabka et al., 2008). Additionally, previous studies have demonstrated that hexacosanal (C26 aldehyde), a chemical constituent of the epicuticular wax layer of barley, strongly induced appressorium formation of *B. graminis* (Leroch et al., 2013); primary alcohols from rice cuticular wax induce appressorium formation in *M. grisea* (Liu et al., 2011). In this study, in a comparison with paraffin and beeswax-coated surface, the spore germination and appressorium formation rates of *A. alternata* were significantly increased on a pear fruit wax extract-coated surface. This finding further confirmed that the physical and chemical characteristics of cuticular wax had an important regulatory role on infection structure formation of phytopathogens.

Protein kinase A is involved in the response process of plant pathogens to epidermal wax physicochemical signals. In the rice blast fungus, appressorium formation is induced upon physical contact with a hydrophobic surface; deletion of the surface hydrophobin *Mpg1* or a transmembrane protein *Pth11* leads to a defect in contact-induced appressorium formation. The surface-induced appressorium defect in all two deletion mutants can be bypassed by the addition of cAMP (Liu and Dean, 1997). These results indirectly verified the role of the cAMP-PKA pathway on the infection structure formation in response to hydrophobic stimulation. In this study, the Δ*AaPKAc* mutant strain significantly reduced the rate of appressorium formation when responding to the physicochemical signal of pear fruit cuticular wax (Figure 3). PKA activity is known to play a major role in the breakdown of storage carbohydrates, e.g., glycogen or trehalose, in both yeast cells and fungal spores (Thevelein, 1988). Therefore, it seems likely that the involvement of PKA in appressorium development and function is largely at the level of glycogen breakdown, which is required for the increase in intracellular glycerol concentration and the subsequent generation of the turgor pressure (Thines et al., 2000). However, deletion of *AaPKAc* did not completely suppress the infection structure information of *A. alternata* on the surface with higher hydrophobicity and different wax (Figure 2). This result suggests that there may be other signal pathways which are also involved in the sensing or response process to the fruit surface and coordinately regulate infection structure information (Kaffarnik et al., 2014; Zhao et al., 2020).

The cAMP-PKA signaling pathway plays significant and conserved roles in the growth of fungus (Turrà et al., 2014; Han et al., 2015). In this study, the Δ*AaPKAc* mutants were reduced in biomass and mycelial growth (Figures 4A,C); this finding agrees with Bormann et al. (2014) who reported that *CPK1* and *CPK2* are apparently required for the growth of *F. graminearum*. Yamauchi et al. (2004) also reported that deletion of *Cpk1* in *C. lagenarium* attenuated the growth rate and caused the defect in conidia germination. This result proved that the PKA catalytic subunit plays a positive role in the growth of different fungi. Simultaneously, most studies have reported that the catalytic subunit of PKA also plays a positive role in conidia production. In *C. higginsianum*, *F. verticillioides*, and *F. oxysporum*, deletion of the PKA catalytic subunit gene significantly reduced conidia production (Jurick and Rollins, 2007; Yang et al., 2016; Zhu et al., 2017). Interestingly, the results presented in this study showed that the deletion of *AaPKAc* increased conidia production of *A. alternata* (Figure 4B), supporting that the regulatory role of the PKA catalytic subunit on regulating conidia production varied with that of fungus. Our work also examined whether the stress tolerance was affected in the deletion of the *AaPKAc* mutant. The result demonstrated that *AaPKAc* had no significant effect on resistance to exogenous H_2_O_2_ (Figures 5A,E). In contrast, in *C. higginsianum*, the *ΔChPKA1* mutants showed increased tolerance to elevated temperatures and exogenous H_2_O_2_ compared with the WT strain (Zhu et al., 2017). In addition, *AaPKAc* plays an antagonistic role in osmotic stress induced by glucose or sorbitol (Figures 5C,D). However, in *C. albicans*, *Tpk1* and *Tpk2* also seem to play opposite roles in regulating stress responses. Deletion of *TPK1* leads to decreased resistance to osmotic and oxidative stresses, whereas deletion of *TPK2* either results in unchanged or increased levels of resistance (Walker et al., 2009). In *S. cerevisiae*, PKA activity leads to a generalized downregulation of oxidative and osmotic stress responses (Fuller and Rhodes, 2012), supporting the diverse roles of the PKA catalytic subunit in different plant fungal pathogens; the effect of *AaPKAc* on the expression levels of stress-related genes will be further explored in the future.

The pathogenicity of Δ*AaPKAc* mutation pear fruits was defective compared with the WT strain (Figure 6), highlighting the importance of *AaPKAc* in the virulence of *A. alternata*. Interestingly, the *ΔcpkA* of *M. oryzae* failed to elicit any visible blast symptoms on barley leaves, but wounding of barley leaves with the micropipette tip helped the *ΔcpkA* to produce WT-like blast lesions in such abraded tissues (Selvaraj et al., 2017). Similarly, both deletion of *CPK1* and *CPK2* genes in *F. graminearum* and deletion of *Cpk1* in *C. lagenarium* caused a defect in pathogenicity (Yamauchi et al., 2004; Hu et al., 2014). Aside from growth and infection structure, the cAMP-PKA signaling pathway is thought to participate in pigment formation and toxin biosynthesis in fungal species (Shimizu et al., 2003; Grosse et al., 2008). Melanin has also been demonstrated to scavenge reactive oxygen species (ROS) and promote survival of macrophages in the host. It has been reported that PKA positively regulates the expression of several genes involved in the melanin biosynthetic pathway (Pukkila-Worley et al., 2005). The data presented in this study showed that the Δ*AaPKAc* mutant significantly enhanced melanin content (Figure 7A). On the contrary, in *Aspergillus fumigatus*, the pkaC1-overexpressing strain led to an increase in melanin content. Because PKA activity showed an increased expression of the *pksP* gene, the polyketide synthase *PksP* is an essential enzyme for the production of dihydroxynaphthalene melanin in *A. fumigatus* (Grosse et al., 2008). Accordingly, it is necessary to further clarify the regulatory mechanism of *AaPKAc* on melanin synthesis in *A. alternata*. Furthermore, previous studies have shown that cAMP-PKA signaling may have pathway- or metabolite-specific regulatory roles in secondary metabolism (Choi and Xu, 2010). PKA negatively regulates the biosynthesis of the secondary metabolite sterigmatocystin in *A. nidulans* (Yu and Keller, 2005), while PKA plays a positive role in the production of thione stearate by regulating the transcription factor *AflR* in *A. nidulans* (Shimizu et al., 2003). The results of the present study showed that in Δ*AaPKAc* the contents of TEN, AME, AOH, and ALT toxins were reduced in *A. alternata* (Figures 7B–E), indicating that *AaPKAc* is required for toxin production in *A. alternata*.

## Conclusion

In conclusion, the cAMP-PKA signal pathway is involved in how *A. alternata* perceives and responds to physicochemical signals from pear fruit cuticular wax. Furthermore, *AaPKAc* is required for pre-penetration, developmental, physiological, and pathological processes, and secondary metabolism of *A. alternata.* We will further explore the molecular mechanism of cAMP-PKA signaling pathway regulating nutritional metabolic pathways of *A. alternata* in the future.

## Data Availability Statement

The original contributions presented in the study are included in the article/Supplementary Material, further inquiries can be directed to the corresponding author/s.

## Author Contributions

MZ, YB, YCL, and DP conceived and designed the experiments. MZ, TW, RL, YH, RM, QJ, and YXL performed the experiments. MZ analyzed the data and wrote the manuscript. All authors have read and agreed to the published version of the manuscript.

## Conflict of Interest

The authors declare that the research was conducted in the absence of any commercial or financial relationships that could be construed as a potential conflict of interest.
