# Supplementary material for: AaPKAc Regulates Differentiation of Infection Structures Induced by Physicochemical Signals From Pear Fruit Cuticular Wax, Secondary Metabolism, and Pathogenicity of Alternaria alternata
Source: Front Plant Sci. 2021 Apr 21;12:642601. doi: 10.3389/fpls.2021.642601 (PMC8096925; doi:10.3389/fpls.2021.642601)
Supplement: Supplementary file 1 [file Data_Sheet_1.docx]

**Supplementary Figure 1**


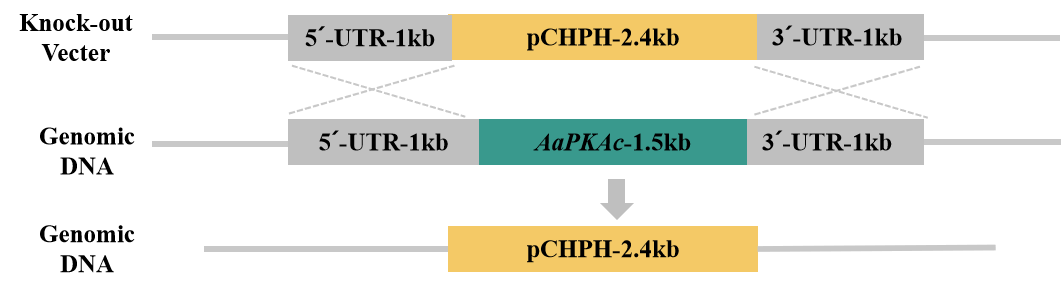


Supplementary Fig. 1 Schematic of the method of *AaPKAc* gene knock-out

**Supplementary Figure 2**


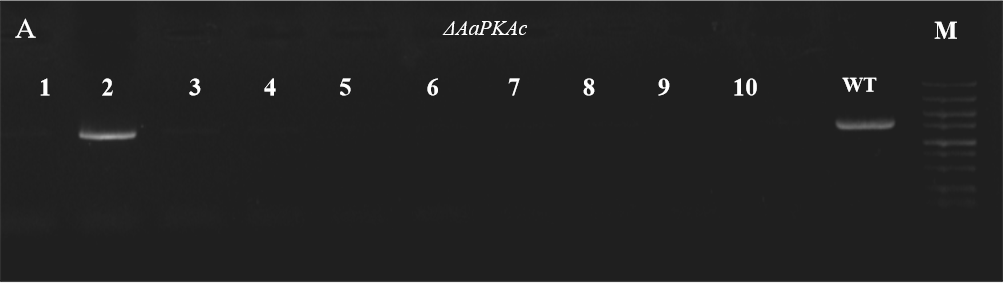
**
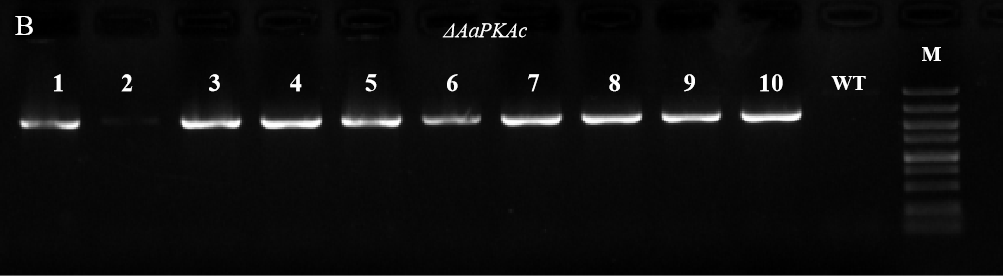
**

Supplementary Fig. 2 Verification of *AaPKAc* target genes in WT strain and *ΔAaPKAc* mutant strain (A) . Verification of *pCHPH* fragments in WT strain and *ΔAaPKAc* mutant strain (B). *AaPKAc* relative expression level in WT strain and *ΔAaPKAc* mutant strain (C). M:5000bp marker.

**Supplementary Figure 3**

**
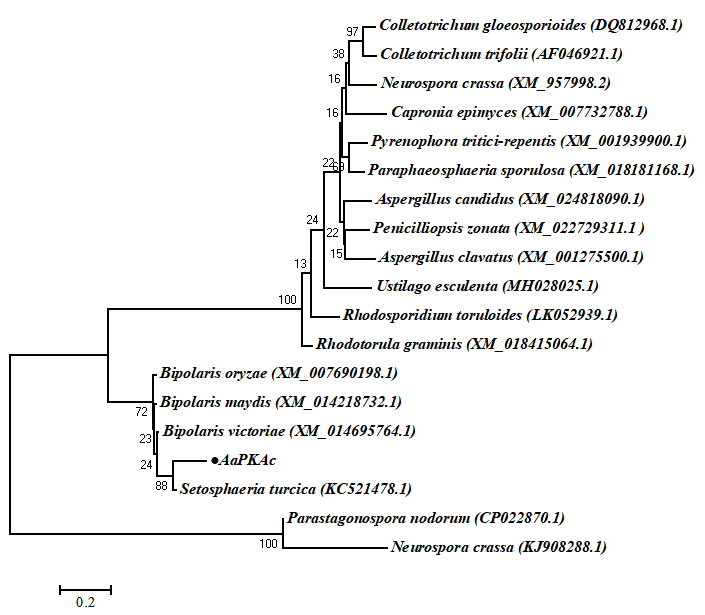
**

**A**

**B**

**
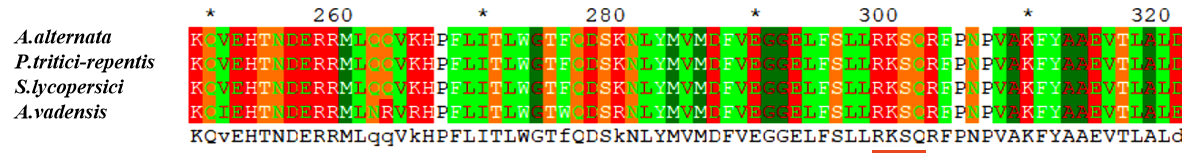
**

Supplementary Fig. 3 Identification of cAMP-dependent protein kinase (PKA) catalytic subunits *AaPKAc* in *A. alternata.* Phylogenetic tree analysis of *AaPKAc* (A). Conserved amino acid sequence alignment of *AaPKAc* with PKA catalytic subunits of *Pyrenophora tritci-repentis*, *S.lycopersici*, and *A. vadensis* (B)*.*

**Supplementary Tables**

| **Supplementary Table 1 Primers used for amplication of *PKAc-up*、*PKAc-down*、*PKAc-c*** | |
| --- | --- |
| **Gene** | **Primer sequences (5' - 3')** |
| *AaPKAc-up-*F | acagctatgaccatgattacGAATTCGAGATACCAGGACCGGAGTA |
| *AaPKAc-up-*R | GATCCCCGGGTACCGAGCTCGAATTCCAGAGTAGGCATTGGAGGG |
| *AaPKAc-down-*F | catgcatggttgcctaactcggcgcgccGACTTCTAAACCCGTAATCG |
| *AaPKAc-down-*R | GACGGCCAGTGCCAAGCTTCggcgcgccGTCGCAAATGTACCCTTC |
| *AaPKAc-c-R* | TCCCCCGGGCTGCAGGAATTCatgcctactctgggcttcct |
| *AaPKAc-c-F* | GGTACCGGGCCCCCCCTCGAGTTAGAAGTCGGGAAATAGGT |
| *AaPKAc-Exp-F* | CGAGACTTGAAGCCCGAGAA |
| *AaPKAc-Exp-F* | GAGTGAAACCGCACAGCATT |

| **Supplementary Table 2 HPLC elution gradient** | | |
| --- | --- | --- |
| **Time（min）** | **mobile phase A** | **mobile phase B** |
| 0 | 90 | 10 |
| 1 | 70 | 30 |
| 2 | 50 | 50 |
| 3 | 10 | 90 |
| 5 | 10 | 90 |
| 5.1 | 90 | 10 |
| 7.1 | 90 | 10 |

| **Supplementary Table 3 Optimized MRM parameters for AOH、AME、 ALT、TEN mycotoxins** | | | | | | | |
| --- | --- | --- | --- | --- | --- | --- | --- |
| **Target compounds** | **Ionization mode** | **Parent ion** | **Qualitative ion** | **keep time** | **Quantitative ion** | **Fragmentation voltage** | **Collision voltage** |
| Altemariol, AOH | ESI^＋^ | 257.0 | 213.0 | 2.37 | 147.2 | 40 | 32 |
| Altermariol monomethylether, AME | ESI^＋^ | 271.0 | 256.0 | 2.85 | 228.0  212.9 | 32 | 20 |
| Allenuene, ALT | ESI^＋^ | 293.1 | 257.2 | 3.33 | 239.1 | 85 | 15 |
| Tentoxin, Ten | ESI^＋^ | 415.2 | 312.3 | 3.66 | 189.0 | 110 | 30 |
